# Increased Delayed Healthcare Among People with Depression and Multimorbidity: Analysis of the 2019-2023 NHIS data

**DOI:** 10.1093/geroni/igaf122.3069

**Published:** 2025-12-31

**Authors:** Arum Lim, Chitchanok Benjasirisan, Soo Hyun Kim, Cheryl Himmelfarb, Patricia Davidson, Binu Koirala

**Affiliations:** Johns Hopkins University School of Nursing, Baltimore, Maryland, United States; Johns Hopkins University, Baltimore, Maryland, United States; Johns Hopkins University, Baltimore, Maryland, United States; Johns Hopkins University, Baltimore, Maryland, United States; University of New South Wales Sydney, Kensington, New South Wales, Australia; Johns Hopkins University, Baltimore, Maryland, United States

## Abstract

Depression is common among individuals living with multimorbidity, causing adverse health outcomes. Although timely healthcare access is crucial to this population due to the burden of illness and the complexity of care, it has not been studied extensively how depression hinders healthcare access. This study aimed to examine the prevalence of delayed healthcare and its association with depression and multimorbidity, stratified by age (18-64 vs. ≥65 years). A cross-sectional analysis was performed using 56,727 individuals’ data from the 2019-2023 National Health Interview Survey (NHIS). Multimorbidity was defined by a self-reported history of two or more physical chronic conditions (e.g., hypertension, diabetes, stroke, cancer), and a self-reported depression history was assessed additionally. Individuals who experienced delays in healthcare over the past 12 months for any reason, including insurance issues, scheduling conflicts, transportation challenges, or costs, were regarded as having delayed healthcare. Social determinants of health, such as race/ethnicity, education, and socioeconomic status, were adjusted in survey-weighted Poisson regression analyses. The mean (±SE) ages of older and younger adults were 75±0.6 and 51±0.1, respectively, with 54% females in both groups. The adjusted prevalence of delayed healthcare was higher in younger adults (22.0%) than in older adults (7.6%). However, older adults with depression had a 2.19 times higher prevalence of delayed healthcare (95% CI: 1.96-2.43), while occurrence was 1.78 times higher in younger adults (95% CI: 1.67-1.91) than those without depression. Depression screening should be administered early for individuals with multimorbidity to identify the risk for delayed healthcare.

